# Auditory Screening in Newborns after Maternal SARS-CoV-2 Infection: An Overview

**DOI:** 10.3390/children10050834

**Published:** 2023-05-04

**Authors:** Virginia Fancello, Giuseppe Fancello, Elisabetta Genovese, Stefano Pelucchi, Silvia Palma, Chiara Bianchini, Andrea Ciorba

**Affiliations:** 1 ENT & Audiology Unit, Department of Neurosciences, University Hospital of Ferrara, 44124 Ferrara, Italy; 2 Department of Otorhinolaryngology, Careggi University Hospital, 50134 Florence, Italy; 3 ENT & Audiology Department, University of Modena and Reggio Emilia, 41121 Modena, Italy

**Keywords:** COVID-19, congenital hearing loss, pregnancy

## Abstract

Background and aim: Several viruses have previously been reported to be responsible for congenital hearing loss; therefore, since the beginning of the SARS-CoV-2 infection pandemic, various reports have investigated a possible link. The aim of this review is to assess the possible link between maternal COVID-19 infection and congenital hearing loss. Methods: This systematic review was performed using PRISMA criteria, searching Medline and Embase databases from March 2020 to February 2023. A total of 924 candidate papers were identified; however, considering the specific selection criteria, only nine were selected for additional analysis. Results: The overall number of children born from mothers infected with COVID-19 during pregnancy identified through this review was 1687. The confirmed cases of hearing loss were 0.7% (12/1688); a description of its nature (sensorineural vs. conductive) is missing in the selected studies, and the follow-up period is variable across the analyzed papers. Surprisingly, a large proportion of false positives were recorded at the first stage of screening, which resulted normal at the re-test. Conclusions: Currently, a correlation between congenital hearing loss and SARS-CoV-2 infection cannot be definitively established. Further studies are desirable to provide additional evidence on this topic.

## 1. Introduction

Hearing loss is the most frequent sensory deficit worldwide and one of the most frequent congenital disorders. The prevalence of this disability ranges from 1 to 3 of every 1000 newborns [1]. Early diagnosis is crucial to establishing prompt rehabilitation and preventing undesirable long-term effects on language development and cognitive abilities. Universal newborn hearing screening programs (UNHS) have previously been proposed for this reason and are widely diffuse.

Maternal infections during pregnancy are well-known risk factors for sensorineural hearing loss. Pregnant women are particularly vulnerable to viral infections, which can result in miscarriage, pre-term birth, and vertical transmission. Furthermore, pregnant women are more likely to have a more severe course of disease, due to the immunological changes caused by pregnancy that can raise the risk of infection [2].

Congenital infections in newborns can have long-term consequences, and asymptomatic babies are also at a greater risk of developing long-term abnormalities. In the past, new viral infections raised concerns of increased instances of sensory deficit in babies; relevant reports are available in the literature. In 2017, a case series of maternal infections were reported during the Middle East respiratory syndrome coronavirus (MERS-CoV) pandemic [3], with a description of full-term delivery without viral transmission. Larger available series described a higher incidence of abortion, stillbirth, prematurity, and congenital malformations in newborns borne from women infected during pregnancy, especially in the first trimester, during the Asian flu pandemic, which occurred in 1957–1958 [4,5]. A study published in 2009 postulated that the Hong Kong flu pre-natal infection (caused by an influenza A (H3N2) virus in the 1968 pandemic) may affect fetal cerebral development [6]. However, although viral infections during pregnancy often lead to congenital hearing loss, no comments on deafness appeared in the above-mentioned studies.

So far, the virus most frequently responsible for congenital hearing loss is Cytomegalovirus (CMV), which accounts for up to 20% of cases [7]. Other reported viral agents responsible for congenital hearing loss are rubeovirus, measles virus, human immunodeficiency virus (HIV), and others [8].

Little is known about the possible features of inner ear damage in case of congenital SARS-CoV 2 infection. During embryogenesis and fetus growth, the development of the inner ear necessitates complex cellular and molecular processes, and a variety of events might alter its growth and/or function. Among these events, a viral infection may cause direct structural damage to cochlear hair cells or spiral ganglion neurons, causing virus-driven apoptosis and, consequently, hearing loss [9]. In particular, a proposed direct mechanism of damage could be related to the angiotensin-converting enzyme 2 (ACE 2) receptor, according to some authors, as these receptors have been found also in the inner ear [10]. Additionally, indirect damage to the inner ear caused by SARS-CoV 2, could be due to perivascular inflammatory infiltration or endothelial dysfunction, with impairment of the inner ear microvessels or stria vascularis [10]. It is likely that there is not one exclusive mechanism of inner ear damage, and most of the pathogenetic potential of SARS-CoV 2 is not completely understood. Furthermore, to date, there are very few animal models reproducing the features of inner ear infections and inner ear damage due to other viruses (i.e., congenital CMV infection); therefore, it is not possible to drive firm conclusions on this issue [10].

Since the World Health Organization declared the SARS-CoV 2 infection pandemic in March 2020, interest in studying possible viral effects on the inner ear gradually increased. Considering the inner ear’s vulnerability to viruses [7,8], several authors described hearing loss, tinnitus, and/or vertigo in relationship with SARS-CoV 2, which also caused long-term morbidity and affected quality of life. The first report concerning a possible association between hearing loss and COVID-19 was published in April 2020; since then, few similar papers have been published, while many authors also investigated the outcome of UNHS programs following maternal viral infection [11].

The aim of this review is to assess a possible link between maternal COVID-19 infection and congenital hearing loss.

## 2. Materials and Methods

This systematic review was performed using English-language studies, for which we searched using papers on the Medline and Embase databases published from March 2020 to February 2023. The most recent literature search was completed in February 2023. The keywords ((“COVID-19” [all fields]) AND “Hearing Loss” [all fields])) were selected to identify the relevant papers.

Inclusion criteria were:
  ○ Established maternal SARS-CoV 2 infection during gestation;
  ○ Evaluation of newborn hearing via at least ABR;
  ○ Studies in English;
  ○ Established follow-up period.
Exclusion criteria:
  ○ Non-confirmed SARS-CoV 2 infection;
  ○ Evaluation of newborn hearing without ABR;
  ○ Insufficient data (i.e., missing outcomes, follow-up period, and/or audiological assessment);
  ○ Studies published in language other than English;
  ○ Studies with duplicated data.

A total of 924 papers were initially identified; however, only 9 described the outcomes of audiological screening in children born to mothers infected by SARS-CoV-2 during pregnancy.

**Figure 1:**
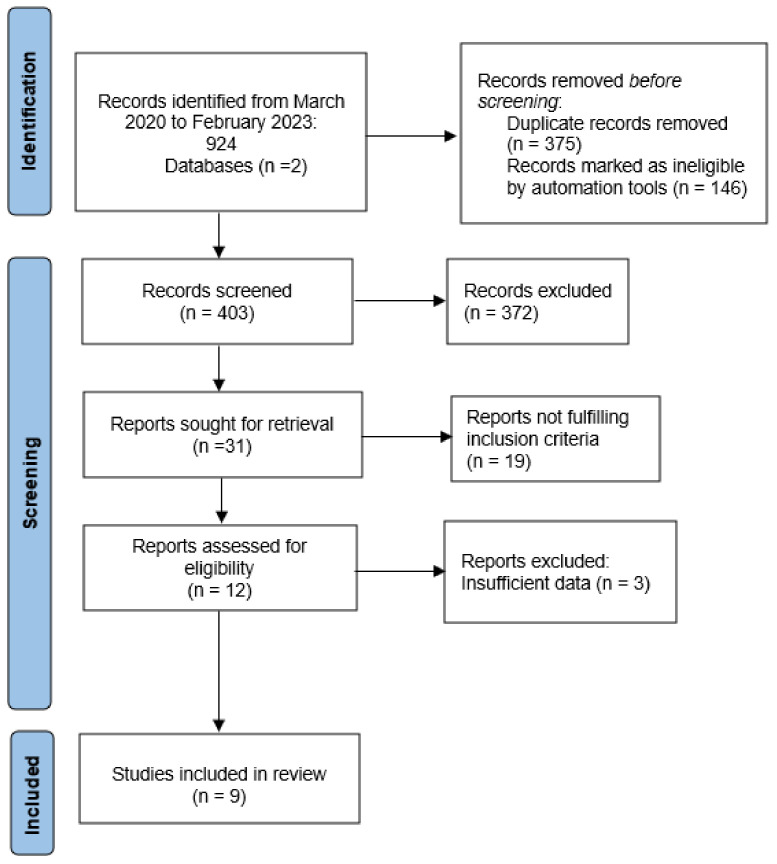
Study selection, as per PRISMA guidelines (http://www.prisma-statement.org/ (accessed on 15 March 2023)).

The review was completed according to the Preferred Reporting Items for Systematic Reviews and Meta-Analysis (PRISMA) guidelines. Figure 1 shows the relative flow diagram.

This research was based on analyzing previously published papers. Therefore, no ethical approval or patient consent was needed. Two investigators (VF and GF) independently assessed the data; information extracted from the included studies were then included in an excel database for further analysis.

The registration number on Prospero was 396723.

## 3. Results

Our search retrieved nine papers, of which seven were retrospective and two prospective. The features of the included papers are reported in Table 1.

The studies were published online in a period ranging from 2021 to early-2023, while included data were collected from early stages of the pandemic to late-2022.

**Figure 2:**
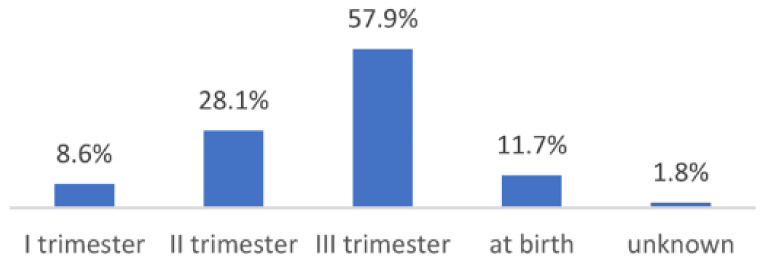
Overall distribution of maternal infection during pregnancy.

**Figure 3:**
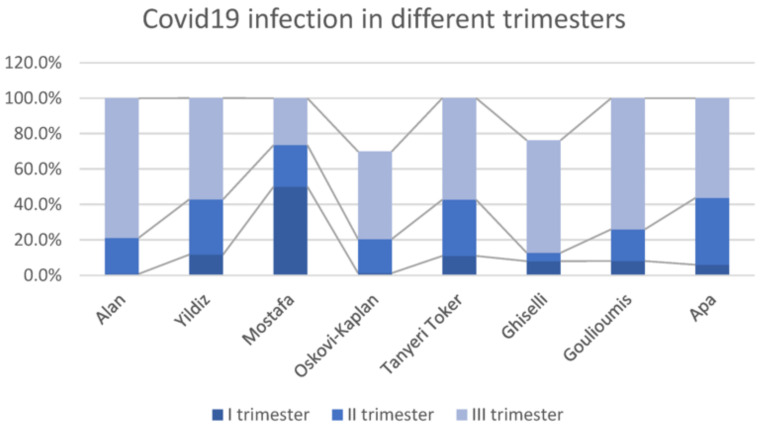
Distribution of maternal infection during pregnancy among studies [12,13,14,15,16,17,19,20].

The timing of maternal infection was analyzed; in the selected studies, the infection occurred during the third trimester or at the time of the delivery in 69.6% of cases (details are illustrated in Figure 2 and Figure 3). This specific finding was shared across all studies except Mostafa et al., while Veeranna et al. did not provide information on this issue.

The maternal age, which was reported in seven over nine studies, ranged from 16 to 33 years old. Overall, the total number of children born from mother infected with COVID-19 during pregnancy was 1687.

Demographics features are summarized in Table 2.

The rate of infants born from COVID-19 infected mothers who had a proven SARS-CoV-2 infection at birth, tested via nasopharyngeal swab, was 0.2% (5/1688).

The follow-up period length was less than one month in six out of nine studies; the audiological evaluation consisted of standard hearing screening tests via means of automated ABR and/or otoacoustic emissions (OAEs). Only three studies performed a full audiological evaluation using threshold ABR, and two of these studies also reported outcomes of tympanometry (see Table 3).

**Figure 4:**
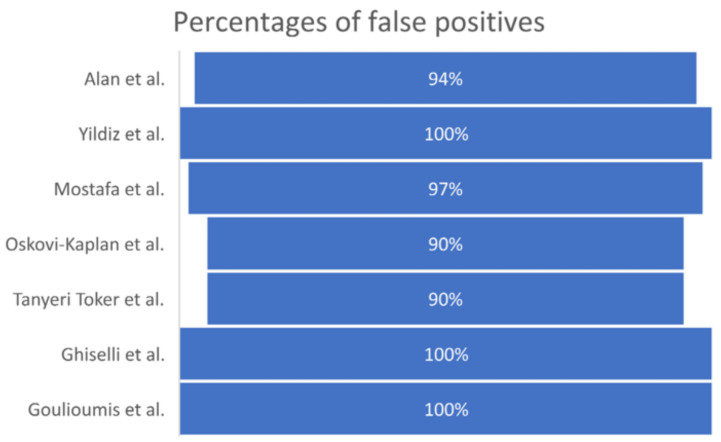
Studies who reported false positive outcomes and their percentages [12,13,14,15,16,17,19].

All the studies reported the results at the first screening and re-test(s) (second or more). The discrepancy between the first and second test results was extremely high, with the majority of screening tests presenting false positives (Figure 4).

Overall, confirmed cases of hearing loss represented 0.7% (12/1688) of all cases for the children born from a mother who was affected SARS-CoV-2 during pregnancy in different trimesters of gestation.

**Figure 5:**
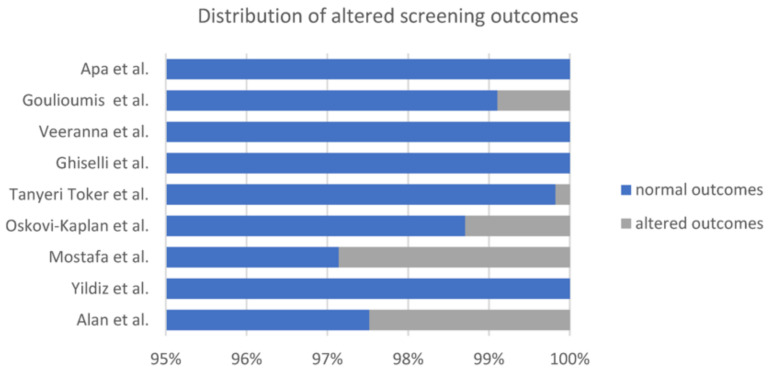
Outcomes at last screening test among different studies [12,13,14,15,16,17,18,19,20].

Figure 5 illustrates the final outcomes of the screening process. Interestingly, the authors who performed a full audiological evaluation (Apa and Ghiselli) reported normal hearing screening results for 100% of newborns, emphasizing the importance of a comprehensive evaluation for both diagnostic and therapeutic purposes; tympanometry in particular is critical for differential diagnosis in case of middle ear effusion, as pointed out in several studies [17,20]. The studies presenting the higher prevalence of altered results were those by Alan and Mostafa [12,14]; in both cases, ABR testing performed to clear the screening results was crucial for a proper assessment, since it confuted the results of the hearing screening.

In addition, most studies do not provide a description of the nature (sensorineural vs. conductive) and level of hearing loss, even if none of the authors mentioned cases of profound SNHL.

Five studies [12,14,15,16,18] compare the outcomes of COVID-19 groups with controls, which are defined as infants born from mothers without a positive COVID-19 test during pregnancy, with different findings. Mostafa, Oskovi-Kaplan, and Tanyeri Toker [14,15,16] did not define any difference between the COVID-19 and control groups, although they reported a more likely “refer” result at the first screening test in the COVID-19 group (which was then not confirmed at the retest) (see also Figure 5).

On the other hand, in his early report, Alan [12] postulated that infants born from COVID-19-infected mothers were more likely to present a refer result at the automated ABR. However, the follow-up period was limited to the first month of life for these children, and the features and levels of hearing loss were not reported.

Veeranna et al. [18] also investigated the ABR threshold for these infants. He described increased absolute latencies of waves III and V, and increased I-V interpeak intervals in the COVID-19 group compared to paired controls. However, DPOAE outcomes and absolute latency of wave I were similar between groups. These findings may suggest a delay in the auditory system maturation; however, longer follow-up periods are required to support this hypothesis.

## 4. Discussion

SARS-CoV-19 affected a large number of people worldwide, including many pregnant women. In adults, neurological disorders are described in up to 80% of severe cases, and the theories about the neurotropism of this new virus are supported by the high degree of chemosensory impairment.

Many theories were suggested to clarify the etiology of neurological symptoms documented in SARS-CoV-2 infection. These theories include hypoxia, immune-mediated damage, coagulative disorders, direct viral injury, or a combination thereof.

The majority of mothers and newborns undertook a routine clinical course, although COVID-19 maternal infection is linked to pregnancy complications, such as increased cases of caesarian section, pre-term births, and higher neonatal intensive care unit (NICU) admission rates [21]. In addition, the SARS-CoV-2 is described as a potential trigger of systemic conditions, such as Guillain–Barré syndrome, which has also been reported in a SARS-CoV-2-infected pregnant woman. According to that report, the patient developed a fast bilateral facial nerve palsy, which is associated with lower extremity paresthesia and audio-vestibular impairment. Despite this complication, the pregnancy proceeded successfully, and a healthy baby was born spontaneously at 40 weeks [22].

The ability of COVID-19 infection to affect the fetus during pregnancy is currently unknown, and, according to the data available, clear evidence of viral intrauterine vertical transmission is not established [21].

The route of the infection to the fetus is the placenta blood; however, perinatally, the infection could also occur via vaginal secretions or breast milk.

Placental contamination is a serious concern, with several described adverse consequences for both the mother and fetus. Histopathological examination performed on the placentas of women who experience COVID-19 during their gestational period revealed signs of vascular malperfusion, intervillositis, perivillous fibrin deposition, and necrosis [23]. The incidence of trans-placental viral transmission is reported as a very rare event, even when the placenta tested positive for SARS-CoV-2, indicating that the placenta may be infected without newborn contamination. [24].

Few small studies address the risk of intrapartum vaginal transmission; however, no clear clinical evidence of the virus’ presence in vaginal secretions exists. [25,26]. Moreover, an increased frequency in cesarian section cases in cases of maternal COVID-19 is described by some authors [15,27]; however, a large multicentric US study does not support suggestions that the prevalence of cesarian section in infected women is because of the high rate of complications [28].

Breastmilk may eventually contain SARS-CoV-2 RNA following a recent infection; however, to date, there is no evidence linking breastfeeding to an increased risk of SARS-CoV-2 infection in babies. According to the literature, proof of infectious SARS-CoV-2 in breast milk does not yet exist. [29].

Since viral infections are reported to be responsible for congenital sensorineural hearing loss, the hypothesis of possible direct hearing damage caused by SAR-COV-2 has been explored since the early stages of the pandemic. Investigations were facilitated via the diffusion of UNHS in the last two decades, alongside technological advances in screening [30]. In the regions where the UNHS program is consolidated, all newborns undergo OAEs or automated ABR; moreover, a two-step screening process, which includes both OAE and ABR, is performed in infants who do not pass the first step or present the risk factors, such as gestational exposure to viruses.

The present review identified studies from different countries and, as expected, most of the data were extracted from hearing screening program databases. The use of OAEs is proven to be a simple, rapid, accurate, and cost-effective tool that provides a non-invasive objective indicator of cochlear function. Both TEOAEs and DPOAEs are reported as first-line screening tools [30].

Celik et al. described a possible congenital audiological impairment, which they specifically related to insufficiency in the medial olivocochlear efferent system, in newborns exposed to SARS-CoV-2 during gestation and only assessed via TEOAEs or DPOAEs [31]. However, any suspected cases of hearing loss must be confirmed via both a threshold ABR and repeated testing at the follow-up stage. In fact, a variable number of false positives may be recorded at OAEs; as reported by some of the authors in the review’s selected studies, OAEs were disproved during the second round of OAE or ABR testing in 96% of cases, while the literature reports false-positive rates for UNHS between 1.8% and 8% [32].

Cases of auditory neuropathy, which are typically characterized by normal OAEs and desynchronization at ABR, were not mentioned in the analyzed series.

Other non-viral risk factors may influence audiological outcomes. At the beginning of the pandemic, we assisted an off-label administration of hydroxychloroquine, a medication with known ototoxic consequences. Later on, the Health Surveillance Agencies, due to the lack of evidence about potential positive effects, restricted its prescription [33]. Its administration was reported in pregnant women by Alan and Ghiselli, though proof of a negative impact on audiological outcomes was not found [12,17].

Furthermore, five of the selected studies presented a control group [12,14,15,16,18]. It was interesting to notice a tendency to compare “refer” results at the first screening test in the COVID-19 group to controls, even though, at re-test, hearing loss was not confirmed. A possible delayed maturation of the auditory pathway was suggested by Veeranna et al. [18], providing an explanation for the high rate of alteration reported at the first screening, which was performed in most studies via means of automated ABR.

One study [12] stated that 20 patients with “refer” results at the first screening were lost in the follow-up period. Especially during the first pandemic waves, the accuracy of screening was influenced by the increased number of dropouts, as reported by other papers [34].

So far, SARS-CoV-2 does not appear to affect infants in the short term [35], and no cases of profound hearing loss related to gestational infection have been described in the literature. However, the late onset of sensorineural hearing loss secondary to gestational infections is an event described in relation to other pathogenic agents, such as cytomegalovirus. Thus, the design of prospective cohort studies in this field could be useful in order to investigate both the long-term hearing and neurodevelopmental outcomes, which are currently unknown.

Another issue could be related to possible effects of COVID-19 vaccination, as massive immunization programs developed after December 2020. No major concerns about the administration of the vaccine to pregnant women arose; hence, after the first phases of the campaign, the vaccination was also offered to this subgroup [36]. Since antibodies (IgG) transmission through the placenta was described to occur in most babies born from mothers who experience the infection during gestation [36], it is likely that maternal SARS-CoV-2 antibodies created by the COVID-19 vaccine could have been transferred from mother to fetus via the placenta, presuming the same mechanism [37]. Consequently, it is possible that the large diffusion of COVID-19 immunization among women of reproductive age could have played a crucial role (i) in protecting the mothers from developing a severe disease, and (ii), eventually, protecting newborns from possible long-term complications related to virus exposure.

Drawbacks: Major limitations of this review are (i) the heterogeneous diagnostic tools used in the screening and (ii) the short-term follow-up period. Moreover, the absence of a description of hearing loss is a major shortcoming; in fact, details on the nature and level of hearing loss were not provided in most of the cases. Differential diagnosis among conditions, such as middle ear effusion, which can be simply evaluated via tympanometry, is crucial for the definition of such impairments.

## 5. Conclusions

Currently, a correlation between congenital hearing loss and gestation SARS-CoV-2 infection cannot be established certainly, as per other viruses [31]. However, during the first screening assessment, especially when it was performed via means of automated ABR, an extremely high rate of false positives was reported, then confuted at the re-test stage.

The long-term impact of COVID-19 on the neurodevelopment of infants with a history of intrauterine virus exposure needs to be further explored. Future prospective studies are desirable to provide additional evidence on this topic.

## Figures and Tables

**Table 1:** Studies included in analysis. (P = prospective study, R = retrospective study, Timing = month/year).

Authors	Ref.	Study	Year	Timing	Country
Alan et al.	[12]	R	April 2021	4/2020–12/2020	Turkey
Yildiz et al.	[13]	R	August 2021	4/2020–5/2021	Turkey
Mostafa et al.	[14]	R	September 2021	11/2020–4/2021	Egypt
Oskovi-Kaplan et al.	[15]	R	November 2021	3/2020–10/2020	Turkey
Tanyeri Toker et al.	[16]	R	February 2022	3/2020–5/2021	Turkey
Ghiselli et al.	[17]	P	February 2022	2/2020–2/2021	Italy
Veeranna et al.	[18]	R	May 2022	3/2021–3/2022	USA
Goulioumis et al.	[19]	R	December 2022	2/2020–6/2022	Greece
Apa et al.	[20]	P	January 2023	11/2020–11/2021	Italy

**Table 2:** Demographics features of selected studies. (“-“ indicates data not available) [12,13,14,15,16,17,18,19,20].

Authors	Newborn COVID-19 Group	Newborn Control Group	Maternal Mean Age (Range)
Alan et al.	118	118	27 years
Yildiz et al.	199	-	30 years (18–43)
Mostafa et al.	34	887	-
Oskovi-Kaplan et al.	458	339	28 years (16–42)
Tanyeri Toker et al.	570	570	28 years (18–42)
Ghiselli et al.	63	-	32 years (21–42)
Veeranna et al.	15	40	-
Goulioumis et al.	111	-	-
Apa et al.	119	-	31 years
Total	1687	1954	(16–43)

**Table 3:** Audiological characteristics of studies. FU = follow-up. ABR = Auditory brainstem response; TEOAE = Transitory evoked otoacoustic emissions; DPOAE = Distortion product otoacoustic emissions; ✔ = Performed [12,13,14,15,16,17,18,19,20].

Authors		FU	ABR	TEOAE	DPOAE	TYMPANOMETRY
Alan et al.	Screening	≤30	✔			
Yildiz et al.	Screening	≤30	✔	✔		
Mostafa et al.	Screening	≤30	✔	✔		
Oskovi-Kaplan et al.	Screening	≤30	✔	✔		
Tanyeri Toker et al.	Screening	≤30	✔			
Ghiselli et al.	Audiological Evaluation	~100	✔	✔	✔	✔
Veeranna et al.	Audiological Examination	~287	✔		✔	
Goulioumis et al.	Screening	≤30	✔	✔		
Apa et al.	Audiological Examination	~90–120	✔	✔	✔	✔

## Data Availability

Not applicable.
